# Adverse Drug Reactions and Expected Effects to Therapy with Subcutaneous Mistletoe Extracts (*Viscum album* L.) in Cancer Patients

**DOI:** 10.1155/2014/724258

**Published:** 2014-01-19

**Authors:** Megan L. Steele, Jan Axtner, Antje Happe, Matthias Kröz, Harald Matthes, Friedemann Schad

**Affiliations:** ^1^Research Institute Havelhoehe, 14089 Berlin, Germany; ^2^Hospital Havelhoehe, 14089 Berlin, Germany; ^3^Institute for Social Medicine, Epidemiology and Health Economics, Charité-University Medical Center, 10117 Berlin, Germany

## Abstract

*Background.* In Europe, mistletoe extracts are widely used as a complementary cancer therapy. We assessed the safety of subcutaneous mistletoe as a conjunctive therapy in cancer patients within an anthroposophic medicine setting in Germany. *Methods.* A multicentre, observational study was performed within the Network Oncology. Suspected mistletoe adverse drug reactions (ADRs) were described by frequency, causality, severity, and seriousness. Potential risk factors, dose relationships and drug-drug interactions were investigated. *Results.* Of 1923 cancer patients treated with subcutaneous mistletoe extracts, 283 patients (14.7%) reported 427 expected effects (local reactions <5 cm and increased body temperature <38°C). ADRs were documented in 162 (8.4%) patients who reported a total of 264 events. ADRs were mild (50.8%), moderate (45.1%), or severe (4.2%). All were nonserious. Logistic regression analysis revealed that expected effects were more common in females, while immunoreactivity decreased with increasing age and tumour stage. No risk factors were identified for ADRs. ADR frequency increased as mistletoe dose increased, while fewer ADRs occurred during mistletoe therapy received concurrent with conventional therapies. *Conclusion.* The results of this study indicate that mistletoe therapy is safe. ADRs were mostly mild to moderate in intensity and appear to be dose-related and explained by the immune-stimulating, pharmacological activity of mistletoe.

## 1. Introduction

Effective treatment of cancer remains one of the biggest challenges to modern medicine. Due to conventional therapies such as chemotherapy and radiation often falling short of their goals, and to patient dissatisfaction concerning adverse effects associated with these treatments, complementary and alternative medicines (CAM) are becoming increasingly popular [1]. Anthroposophic medicine (AM), founded in the 1920s by Rudolf Steiner and Ita Wegman, is a person-centred medical approach which combines conventional medicine with the use of CAM remedies and specialised therapies, such as physical and artistic therapies [2]. AM uses an integrative approach to treat cancer, focusing not only on elimination of pathological entities (conventional therapies), but also activating salutogenetic resources by using European mistletoe extracts (*Viscum album *L.), and other therapies, with the aim of improving health related functions or preventing further disease [2]. Mistletoe therapy is amongst the most frequently used complementary treatments by cancer patients in Europe [3]. In 2003 more than 18 million defined daily doses were prescribed in Germany [4].

Mistletoe extracts have been shown to kill cancer cells in vitro and can stimulate immune system cells both in vitro and in vivo [5–7]. Two components of mistletoe, namely, viscotoxins and lectins, have been shown to be largely responsible for these effects [7–9]. Reports on the clinical efficacy of mistletoe therapy have been conflicting and systematic literature reviews often criticise studies for poor design [10, 11]. Although an effect of mistletoe on tumour shrinkage in vivo or overall survival might remain to be proven more thoroughly, a growing number of studies indicate beneficial effects on quality of life of cancer patients and reduction of adverse drug reactions (ADRs) associated with conventional cancer treatments [12–17]. Until recently, however, the safety of mistletoe therapy itself and CAM medications in general has been largely overlooked. Reporting of CAM-related ADRs was neglected in the past due to the belief that CAM are “natural” and therefore safe [18]. Furthermore the possibility of harmful interactions between CAM and conventional medications has been discussed [19, 20]. A number of recent controlled clinical trials, mostly using low doses of one type of mistletoe, have focused on tolerability and safety of mistletoe therapy [16, 21, 22]. The conclusions of these studies ranged from “no safety concerns” to “excellent tolerability” [16, 22]. In clinical practice, however, patients generally receive a wide range of doses and mistletoe products, often in conjunction with conventional therapies. Therefore, the aim of this study was to assess the safety of subcutaneous application of mistletoe extracts as a conjunctive therapy in cancer patients, under standard clinical practice within an AM setting in Germany. This involved detection and classification of ADRs according to internationally accepted guidelines to describe the frequency, causality, severity, and seriousness of ADRs attributed to mistletoe therapy. Additionally, potential risk factors, dose relationships, and drug interactions were investigated in order to identify populations with increased susceptibility to experiencing an ADR to mistletoe.

## 2. Methods

### 2.1. Study Design and Data Sources

The present study was designed as a multicentre, observational study within the Network Oncology (NO), a conjoint clinical registry of German hospitals and outpatient practitioners specialised in AM [23, 24]. The NO was established with the aim of developing a database to allow evaluation of integrative therapeutic interventions in AM. Qualified medical documentation officers systematically extract basic patient information, cancer diagnoses, therapies, adverse events, and disease progress from patient files, and record data using the QuaDoSta (Quality management, Documentation, and Statistics) software that was developed at Havelhoehe Research Institute [25]. Along with conventional therapies, such as chemotherapy, radiation, and surgery, the NO documents detailed information about mistletoe therapies received, including dosage data, therapy start and end dates, and related adverse events. Data are entered through a web-based interface comprising checklists and drop down menus, which are linked to regularly updated catalogues (e.g., International Classification of Diseases-10: ICD-10) to ensure uniform data [26]. Additionally, regular training and a telephone support are provided to maintain and enhance data quality. All analyses were conducted and figures created with R version 2.15.1 [27].

### 2.2. Selection of Patient Data

Patient data recorded in the NO database between June 2009 and June 2012 were analysed by trained investigators. Data was checked for completeness and plausibility, corrected if necessary, and subjected to inclusion/exclusion criteria. Only patients with a valid identification number, birth date, gender, cancer diagnosis date, ICD-10 code, and at least a start or end date for mistletoe therapy were included in the final analyses. Descriptive statistics were used to describe patient demographics and the Wilcoxon rank sum (*W*) was used to test for differences between groups. The disease stage of patients at diagnosis was described according to Union for International Cancer Control (UICC) staging. The relationship between age and UICC stage at diagnosis was assessed by a one-way analysis of variance. Types of mistletoe and conventional therapies received are summarised.

### 2.3. Assessment and Classification of Expected Effects and Adverse Drug Reactions

All mistletoe-related expected effects and adverse events reported by physicians were assessed by the study centre. Expected effects were local reactions <5 cm and increased body temperature <38°C. Local reactions and increased temperatures beyond these desired levels, and all other adverse events, were assessed as suspected mistletoe ADRs if a causal relationship between mistletoe and an event was described by physicians as at least a reasonable possibility. Causality of suspected ADRs was then assessed by the investigators according to the World Health Organization Uppsala Monitoring Centre (WHO-UMC) causality assessment system [28]. Expected effects and ADRs with “possible,” “probable,” or “certain” causality were classified as MedDRA 15.0 preferred terms (developed under the auspices of the International Conference on Harmonization: ICH) and grouped by organ manifestation (WHO SOC-code) [28, 29]. ADRs were evaluated in terms of severity (grades 1 to 5) according to the Common Terminology Criteria for Adverse Events (CTCAE) v4.0 [30] and designated as serious or nonserious according to ICH guidelines [29]. The numbers of expected effects and ADRs experienced per person were determined and the management and outcome of ADRs are summarised.

### 2.4. Predictive Factors for Experiencing at Least One Expected Effect or Adverse Drug Reaction

A logistic regression model was developed to evaluate odds ratios (OR) and their 95% confidence intervals (CI) to predict expected effects or ADRs based on potential influencing factors. The regression model included age, gender, and UICC stage. The incidences of ADRs in response to very low, low, moderate, and high doses of mistletoe were compared in order to determine whether experiencing an ADR was dose-dependent. Additionally, the incidences of ADRs during mistletoe therapy with concurrent conventional therapies (chemotherapy, targeted therapies, hormone therapy, bisphosphates, radiation and surgery) were determined in order to see whether receiving mistletoe and a conventional therapy concurrently increased the risk of experiencing an ADR.

## 3. Results

### 3.1. Patient Characteristics and Summary of Conventional Treatments Received

**Figure 1:**
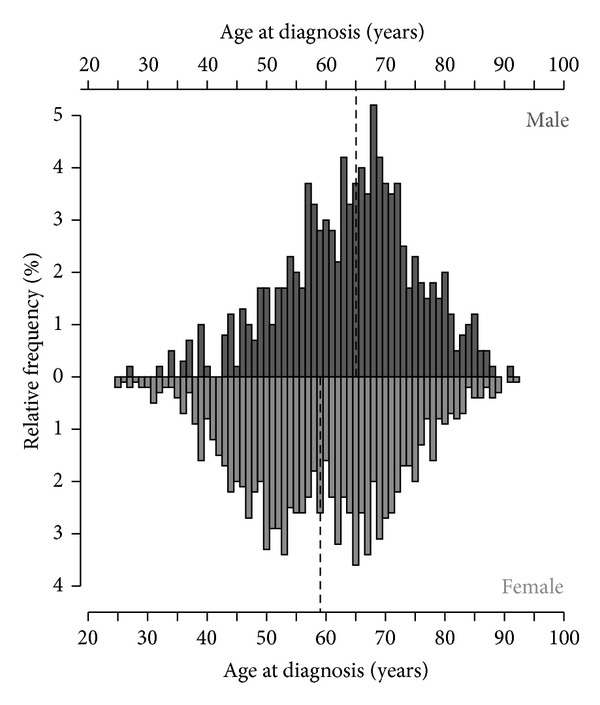
Age of cancer patients treated with mistletoe extracts with respect to gender. The dashed lines denote the respectable median age of both genders.

**Figure 2:**
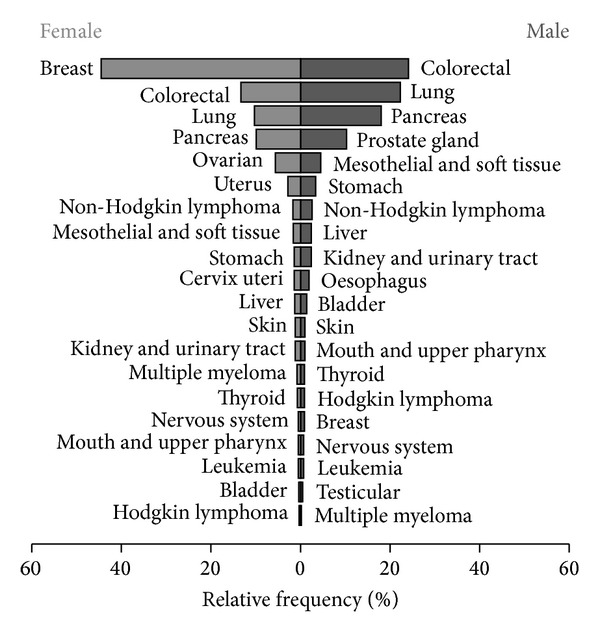
Relative frequency of the most common cancer types in patients treated with mistletoe extracts with respect to gender.

The medical records of 2,131 cancer patients from three hospitals, one rehabilitation clinic, and seven outpatient oncologists in Germany that received mistletoe extract therapy were analysed. The data of 208 patients were excluded due to not meeting the inclusion criteria (patients only received mistletoe extracts by application types other than subcutaneous: *n* = 191; no start or end date for mistletoe therapy recorded: *n* = 17). The final analysis was performed on 1923 cancer patients made up of 1,325 (68.9%) females and 598 (31.1%) males treated with mistletoe extracts by subcutaneous application between July 1, 1999 and June 30, 2012. The age of female patients at first diagnosis ranged from 25 to 92 years and for male patients ranged from 27 to 91 years. The median age of male patients (65 years) was six years older than that of female patients (59 years) (*W* = 298932, *P* < 0.001). The age distribution of patients, with respect to gender, is shown in Figure 1. Breast cancer (582 patients) was by far the most common cancer entity, followed by colorectal, lung, and pancreas (313, 264, and 233 patients, resp.). The relative frequencies of the most common cancer entities in the treated population, with respect to gender, are shown in Figure 2. At the time of diagnosis, 23 patients (1.2%) had a UICC stage of 0, 292 (15.2%) were stage I, 431 (22.4%) were stage II, 369 (19.2%) were stage III, and 491 (25.5%) were stage IV. The UICC stage at diagnosis was not known for 317 (16.5%) patients. There was a highly significant relationship between increasing UICC stage and increasing age at diagnosis (*P* < 0.001). In addition to mistletoe therapy, chemotherapy was used in 55.6% of patients, hormonal therapy in 17.0%, targeted therapies (i.e., monoclonal antibodies, proteasome inhibitors, signal transduction inhibitors) in 16.6%, and bisphosphonates in 5.8%. Radiation therapy was used in 35.6% of patients and 82.4% of patients had at least one surgery.

### 3.2. Mistletoe Extract Therapy

**Figure 3:**
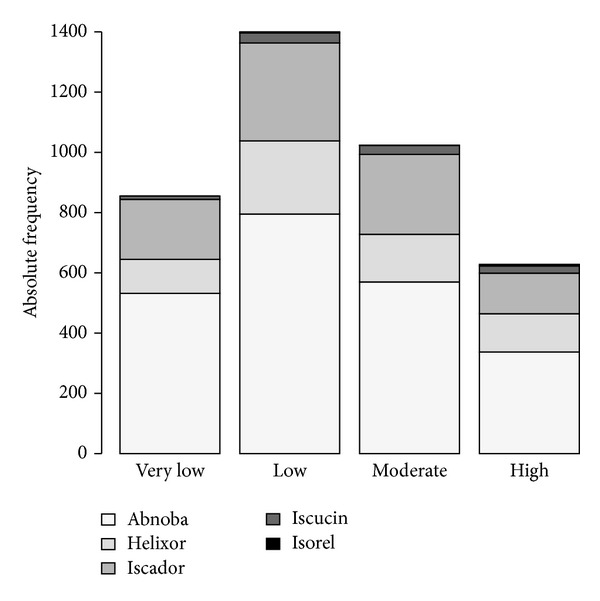
Number of patients that received subcutaneous applications of mistletoe extracts at different dose levels. Dose ranges for each level are shown in Table 1.

The median length of time between first diagnosis and the start of mistletoe therapy was 3.6 months (CI = 2.9–4.0). Total periods of time for which patients received mistletoe therapy ranged from one day to 11 years. The median length of therapy was 4.6 months (CI = 4.1–5.3) and the mean length was 1.1 years (standard deviation of 1.6 years). Generally, patients received mistletoe injections three times per week. Mistletoe extracts from Abnoba were the most frequently used (1315 patients), followed by Iscador (444 patients), Helixor (323 patients) and Iscucin (67 patients). Rarely used mistletoe extracts were from Lektinol (12 patients), Isorel (5 patients), and Eurixor (1 patient). The most common mistletoe host trees were ash (fraxini = 789 patients), apple (mali = 561 patients), oak (quercus = 357 patients), pine (pini = 268 patients), and spruce (abietis = 195 patients). Other host trees were maple (aceris), birch (betulae), elm (ulmi), willow (salicis), almond (amygdali), poplar (populi), hawthorn (cratagi), and linden (tiliae) (146 patients). Although the dose ranges of different mistletoe products vary markedly, doses of mistletoe extracts received by patients can be summarised by classifying doses as very low, low, moderate, or high depending on the product, as shown in Table 1. The numbers of patients that received very low, low, moderate, or high doses of different types of mistletoe extracts are shown in Figure 3. As per the summary of product characteristics (SPC) for each of the products, mistletoe extract therapy mostly started with a very low or low dose of mistletoe extract, increasing to a moderate or high dose over time [31–33].

### 3.3. Expected Effects and Adverse Drug Reactions Attributed to Mistletoe Extract Therapy

Of the 1923 patients treated with mistletoe extracts, 414 (21.5%) patients experienced either an expected effect or an ADR. Of 691 events in total, 666 (21.2% of total patients) were local or temperature related reactions and 25 (0.9% of total patients) were systemic reactions (blood glucose/pressure changes, chills, diarrhoea, fatigue, headache, malaise, nausea, vomiting, syncope, rash, urticaria, and pruritus). Expected effects made up the majority of reactions, with 283 patients (68.4% of patients experiencing a reaction and 14.7% of total patients) reporting 427 expected effects (61.8% of all events). These consisted of local reactions <5 cm (erythema = 44.7%, induration = 5.6%, burning sensation = 4.7%, lymphadenopathy = 1.6%, eosinophilia = 0.2%) and increased body temperature <38°C (43.1%).

A total of 264 ADRs were documented in 162 (8.4%) cancer patients treated with mistletoe extracts (Table 2). ADRs were classified as having possible (42.1%), probable (53.4%), or certain (4.5%) causality according to the WHO-UMC causality assessment. The majority (92.4%) of ADRs fell under the system organ class of general disorders and administration site conditions. These consisted of local reactions >5 cm (injection site erythema, swelling, and urticaria) and increased body temperature >38°C, along with chills, fatigue and malaise. The remaining 7.6% of ADRs were made up by diarrhoea, nausea, vomiting, headache, increased blood glucose, decreased blood pressure, rash, syncope and generalised pruritus, and urticaria.

Of the 264 ADRs, nearly all were rated as being of mild/stage I (50.8%) or moderate/stage II (45.1%) intensity, according to the CTCAE. Increased body temperature between 39°C and 40°C made up 87.4% of the ADRs rated as having moderate intensity, with local reactions >5 cm making up 7.6% and other reactions (chills, diarrhoea, vomiting, pruritus, and urticaria) making up the remaining 5.0%. Only 11 ADRs (4.2% of ADRs), experienced by 11 patients (0.6% of total patients), were judged to be severe/stage III according to CTCAE. These were eight patients with increased body temperature >40°C (less than 24 h), one patient with severe injection site swelling, one patient with generalised urticaria, and one patient with syncope. Therapy was immediately stopped for these patients and all of the patients made full recoveries. There were no life threatening/stage IV ADRs or deaths/stage V ADRs related to mistletoe therapy. Although three ADRs in two patients were reported as serious by physicians, on closer inspection by the investigators it was determined that one of the ADRs (injection site swelling) was severe but not serious according to the ICH E2A guideline. Furthermore, after consulting the original medical records of one patient who experienced two serious ADRs (generalised pruritus, and urticaria), it was discovered that the ADRs were most likely caused by penicillin and not mistletoe. Therefore no serious ADRs were attributed to mistletoe therapy.

**Figure 4:**
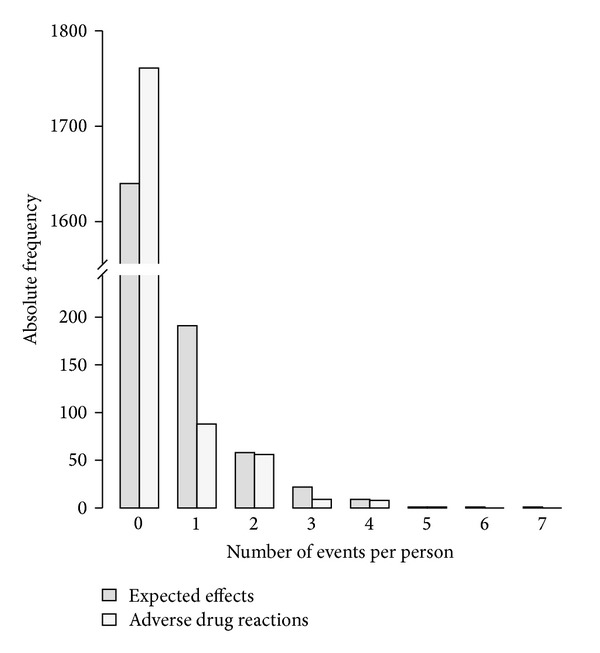
Number of expected effects or adverse drug reactions to subcutaneous mistletoe therapy per patient. Dark grey = expected effects; light grey = adverse drug reactions.

The number of expected effects per patient ranged from zero to seven. Detailed numbers are presented in Figure 4, along with the number of ADRs per patient which showed a similar pattern ranging from zero to five. More than half of the patients experiencing an expected effect or an ADR had only one.

### 3.4. Management and Outcomes of Adverse Drug Reactions

Most ADRs (71.6%) did not require any type of treatment or intervention. For some ADRs, the mistletoe dose was reduced (7.6%) or the product was changed (6.8%). Therapy was stopped in response to only three ADRs (diarrhoea, injection site swelling, local reaction) and two local reactions required dressings. Seven ADRs were treated with AM remedies. These were calcium quercus for injection site urticaria, Combudoron gel for two local reactions, Gelsemium for two cases of pyrexia (one intravenous and one subcutaneous application), Nux vomica for vomiting, and Thrombocutan gel for injection site erythema (initiated by the patient). Only one patient with urticaria required treatment with a conventional medication (intravenous H2 receptor antagonist). Intervention data was missing for 24 (9.1%) patients. Outcome data revealed that 237 (89.8%) patients had “completely recovered,” four (1.5%) patients had “not yet recovered” but were expected to make a full recovery, and data was missing for 23 (8.7%) patients.

### 3.5. Logistic Regression Analysis of Expected Effects and Adverse Drug Reactions

Logistic regression analyses were performed in order to determine potential influencing factors for experiencing an expected effect or an ADR while receiving mistletoe extract therapy. Gender, age, and UICC stage were assessed as variables for predicting expected effects (local reactions <5 cm and increased body temperature <38°C) or ADRs. The results from both analyses are presented in Table 3. It was not possible to include mistletoe dosage in these analyses due to the complexity of the dosage data (i.e., patients often received many different doses and types of mistletoe during their therapy and may have reacted to a certain dose on one occasion, but not on another). The number of patients analysed by logistic regression was reduced from 1923 to 1606 due to the UICC stage at diagnosis being unknown or not applicable for 317 patients. When expected effects were considered (238 patients out of 1606 patients), females were more likely to experience a reaction (OR = 1.61, CI = 1.11–2.39, *P* = 0.015), while older age (OR = 0.97 per year, CI = 0.96–0.99, *P* < 0.001) and increasing UICC stage (UICC I: OR = 0.39, CI = 0.16–0.97, *P* = 0.038; UICC II: OR = 0.39, CI = 0.16–0.95, *P* = 0.033; UICC III: OR = 0.26, CI = 0.11–0.65, *P* = 0.003; UICC IV: OR = 0.15, CI = 0.06–0.39, *P* < 0.001) were associated with less reactions. No risk factors were identified for ADRs (133 patients out of 1606 patients). Based on the rule of ten events per variable [34–36], there were too few events to accurately analyse potential risk factors for more specific types of ADRs (e.g., severe or systemic ADRs).

### 3.6. Relationship between Mistletoe Dose and Incidence of Adverse Drug Reactions

**Figure 5:**
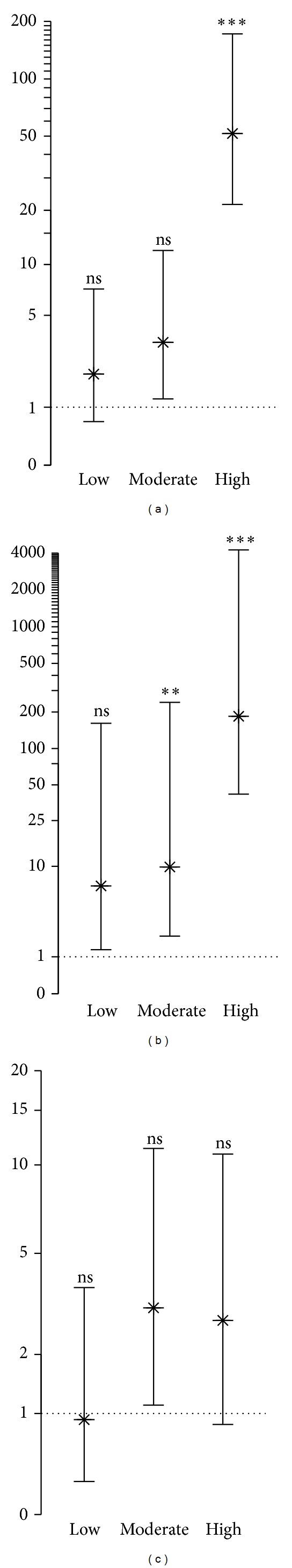
Odds ratios with 95% confidence intervals for the effect of mistletoe dosage on relative frequencies of (a) all ADRs, (b) pyrexia and injection site ADRs only, and (c) other ADRs (diarrhoea, nausea, vomiting, chills, fatigue, malaise, headache, increased blood glucose, decreased blood pressure, pruritus, rash, urticaria, and syncope). The relative frequencies of ADRs in response to low, moderate and high doses of mistletoe were compared to the relative frequency of ADRs that occurred in response to very low dose mistletoe. ns = not significantly different (*P* > 0.05/3), significance levels: **P < 0.01/3, ***P < 0.001/3. Note that normally accepted significance levels are divided by three in accordance with the Bonferroni correction for multiple comparisons.

In order to investigate the relationship between increasing doses of mistletoe extracts and incidence of ADRs, doses were divided into 4 levels: very low, low, moderate, and high. The number of ADRs that occurred at doses within each level was divided by the total number of patients that received mistletoe extracts within each dose level. There was a highly significant relationship between dose level and the incidence of ADRs (Figure 5). Only 0.5%, 1.0% and 1.6% of patients who received very low, low, and moderate doses of mistletoe extracts experienced an ADR at those dose levels. On the other hand, 20.4% of patients who received high doses of mistletoe extracts reported an ADR. This relationship was also seen when only pyrexia and injection site ADRs were considered (very low = 0.1%, low = 0.9%, moderate = 1.3%, high = 20.0%). There was no significant dose-dependent relationship observed for other ADRs however (diarrhoea, nausea, vomiting, chills, fatigue, malaise, headache, increased blood glucose, decreased blood pressure, pruritus, rash, urticaria, and syncope; very low = 0.5%, low = 0.4%, moderate = 1.5%, high = 1.3%). The odds ratios and 95% confidence intervals for the effect of mistletoe dosage on relative frequencies of all ADRs, pyrexia and injection site ADRs only, and other ADRs are shown in Figure 5. Of the 11 severe ADRs (pyrexia x 8, injection site swelling, generalised urticaria, syncope), all occurred in response to high doses (10–20 mg) of mistletoe extracts, apart from the injection site swelling which occurred in response to a moderate dose (0.2 mg).

### 3.7. Incidences of ADRs during Mistletoe Therapy with Concurrent Conventional Therapies

To see whether the frequency of ADRs increased during concurrent therapy with mistletoe extracts and a conventional therapy, the numbers of ADRs that occurred during different therapy crossovers were determined. The results presented in Table 4 show lower incidences of ADRs during therapy crossovers (chemotherapy: 2.41%, targeted therapies: 0.83%, hormone therapy: 0.88%, bisphosphates: 0%, radiation therapy: 2.42%, and surgery: 1.11%) compared to the overall incidence of ADRs (8.42%) recorded in Table 2.

## 4. Discussion

In the present study we assessed expected effects and ADRs in 1923 cancer patients that received subcutaneous mistletoe applications. Expected effects, classified as local reactions <5 cm and increased temperature <38°C, were reported in 14.7% of patients and ADRs were reported in 8.4% of patients. Our results are closely comparable with the published literature, both in terms of incidence and types of ADRs [13, 37, 38]. A retrolective, pharmacoepidemiological cohort study by Bock et al. [37] found that 0.8% of breast cancer patients treated with mistletoe extracts (Iscador) had a systemic reaction and 17.3% experienced local reactions at the injection site, sometimes with mild fever. Our finding that 0.9% of patients experienced a systemic reaction is almost identical. It is not clear whether local reactions <5 cm and increased temperature <38°C were included as ADRs in the analysis by Bock et al. Since expected effects were not discussed in the publication, however, we can assume that there was no cutoff point and that all observed local and fever reactions were recorded as ADRs. In the current study, if we include expected effects along with ADRs, 21.2% of patients experienced local (plus pyrexia) reactions compared to 17.3% as reported by Bock et al. This difference might be explained by the fact that our study specifically concentrated on recording mild reactions (expected effects) such as redness and slight increases in body temperature, in addition to more obvious ADRs. These results are also similar to the results of a systematic review, which included 18 clinical trials examining mistletoe preparations, and reported incidences of 1.6% and 15.9% for systemic and local reactions, respectively [13].

While 8.4% of patients experienced a suspected ADR, which is described as “common” according to the Council for International Organizations of Medical Sciences guidelines [39], only 0.6% of patients had a severe (grade III) suspected ADR, making severe reactions “uncommon.” Severe reactions were mostly pyrexia >40°C which lasted less than 24 h. There were no life threatening (stage IV) ADRs and all reactions were nonserious. ADRs were mostly general disorders and administration site conditions, with other reactions (diarrhoea, nausea, vomiting, headache, increased blood glucose, decreased blood pressure, rash, syncope and generalised pruritus and urticaria) making up only 7.4% of ADRs. Of the recorded ADRs, all have been reported previously by the Federal Institute for Drugs and Medical Devices of Germany (Bundesinstitut für Arzneimittel und Medizinprodukte, www.bfarm.de) and all except for decreased blood pressure, syncope, and increased blood glucose are listed in the SPCs of the products [31–33]. Each of these three suspected ADRs occurred once only and all were described as having “possible” causality. Mistletoe extracts have been shown previously to possess hypotensive activity [40]. In Wistar rats injected with mistletoe extracts, arterial blood pressure was significantly decreased and hexocycline, a selective antagonist of muscarinic receptors, blocked this effect. As with any hypotensive drugs it is possible that an excessive decrease in blood pressure could lead to fainting (syncope). Only one case of possible mistletoe therapy related syncope was found in the literature in a female patient who received high dose mistletoe at six o'clock in the evening, with the intention of inducing pyrexia [41]. The patient collapsed upon getting out of bed in the early hours of the morning (between two and three o'clock) with the result of bruises and a head laceration. It is not clear however whether mistletoe was responsible for the collapse, or if it was a disease-related or spontaneous event [41]. The only studies that were found indicating mistletoe induced changes in blood glucose levels, demonstrated hypoglycaemic effects [42, 43], making it a high possibility of this suspected ADR not actually being related to mistletoe.

Attribution of causality of ADRs is a complex task, often because patients can be receiving multiple therapies at once and might also experience disease-related symptoms that could be confused for ADRs. We classified the recorded ADRs as having “possible,” “probable,” or “certain” causality according to the WHO-UMC causality assessment. Our findings of 42.1% possible, 53.4% probable, and 4.5% certain ADRs correspond with other published findings, which range from 44.4% to 53.2% for possible, 34.0% to 49.8% for probable and 3.3% to 17.7% for certain ADRs [44–46].

A somewhat controversial topic concerning classification of ADRs is the concept of what constitutes an “adverse” event, and what is actually an expected, or even desired effect. Mistletoe therapy is thought to benefit cancer patients through the modulation of cellular and humoral immune responses [47, 48]. Mild local reactions at injection sites and increases in body temperature (<24 h) are frequent observations following applications, and according to product information, are an important part of dose finding strategies during mistletoe therapy [31–33]. These reactions are not considered by physicians as ADRs, but rather as positive indicators of expected pharmacological activity and immune system stimulation. Furthermore, the effect of increasing body temperature and the associated “feeling of warmth” is thought to benefit cancer patients, who often feel cold due to a loss of thermoregulation and reduced temperature amplitudes, especially after chemotherapy and in breast cancer patients [49–51]. Interestingly, significant survival benefits have been achieved in cancer patients treated with hyperthermia in combination with radiation and/or chemotherapy [52–56]. A review by Peer et al. [57] summarized the recent literature identifying complex effects of temperature on immune cells and potential cellular mechanisms by which increased temperature may enhance immune surveillance and tumour control. Although further research is required to better understand the role of body temperature regulation during tumour development and treatment, the temperature boosting property of mistletoe might indeed be a beneficial effect. As a result of differences in the interpretation of what constitutes an ADR, it is not surprising that data regarding incidences of mistletoe-induced ADRs ranges widely. One systematic review on adverse events during mistletoe therapy revealed that incidences ranged from 0.9% to 43% for local reactions and from 0.8% to 4% for systemic reactions [58].

Based on logistic regression results, young to middle-aged females with early-stage cancer were the most likely to experience an expected effect. An identical outcome, in terms of young to middle-aged females reporting the highest incidence of events, was found in a study which assessed the age and gender distribution of suspected ADRs to a variety of newly marketed drugs in general practice in England [59]. A prospective multicentre study based on intensive pharmacovigilance involving 2,371 patients also found a higher risk of ADRs among female subjects compared to males [60]. Although several explanations such as gender-specific pharmacokinetic and pharmacodynamic behaviour of drugs were investigated by Zopf et al. [60], a reason for the gender difference remains unclear but is possibly related to a higher rate of autonomic dysregulation in females [61]. As expected, increasing age at diagnosis was highly correlated with increasing cancer stage (*P* < 0.001). It is well known that cancer and conventional cancer treatments (chemotherapy and radiation) can suppress the immune system [62, 63]. Therefore, it makes sense that patients with late-stage cancer (who were also generally older and had received more chemotherapies/radiation) experienced less expected effects, which are related to stimulation of the immune system, than patients with early-stage cancer. Interestingly, there were no risk factors identified for experiencing an ADR.

The results of our investigation into the relationship between increasing doses of mistletoe extracts and incidence of ADRs suggest that ADRs that are related to the expected pharmacological action of mistletoe, such as local inflammatory reactions and pyrexia, were dose-related. The relationship between dose and other reactions (e.g., diarrhoea, nausea, and vomiting), which are not obviously related to the expected pharmacological action of mistletoe, was less clear. It is important to note however that the low frequency of these reactions makes it difficult to draw any conclusions. Whether specific mistletoe products or host trees were associated with an increased risk of experiencing an ADR was outside the scope of this study but will be investigated in the future.

Finally, the incidence of suspected ADRs to mistletoe injections decreased rather than increased when mistletoe therapy was combined with conventional therapies such as chemotherapy, hormone therapy, targeted therapies, bisphosphates, radiation therapy, and surgery. Importantly, studies have shown that the pharmacokinetics of gemcitabine, an antimetabolite chemotherapeutic agent, are not affected when combined with mistletoe therapy [64, 65]. The reduction in mistletoe ADRs during cotherapy with conventional drugs was probably due to the immunosuppressing nature of many of these therapies [62, 63], providing further evidence that mistletoe extracts are safe for use in cancer patients. Additionally, it has been reported previously that not only is conjunctive therapy with mistletoe extracts safe, it can also reduce the frequency and severity of chemotherapy ADRs [16, 66].

The present study assessed a heterogeneous group of patients in terms of demographic, tumour entities, disease stage, and varied doses, types, and frequencies of subcutaneous mistletoe applications and concurrent therapies. Though potentially limited in terms of too many factors being involved that could have influenced the outcome, our study provides an accurate picture of the clinical use of subcutaneous mistletoe therapy within the NO and its overall safety. A weakness of observational studies is missing or erroneous information for some patients. This problem was addressed by only including patients with complete and valid core data (birth and diagnosis dates, gender, an ICD-10 code, either a start or end date for mistletoe therapy and the product name). For the 1923 eligible patients, all ADRs were reported as long as a valid date was listed. Other limitations of this study include the possibility of underreporting by physicians, especially of expected effects and mild ADRs, and less frequent reporting of ADRs by ambulatory patients due to reduced contact with physicians. On the other hand, it is possible that ADRs were overreported since there was no untreated patient group for which disease-related or spontaneous events could be controlled for. A study by Reidenberg and Lowenthal [67] revealed that even when healthy, young, non-medicated subjects were asked to document everyday symptoms for three days, 41% of patients reported fatigue, 15% a headache, and 10% muscle pain, among others, indicating a high probability of false positive findings in our clinical findings. Furthermore, only conventional or AM interventions related to cancer treatment were recorded for patients in the present study, meaning it is possible that reactions to external interventions (e.g., self-medication, herbal remedies) could have influenced our results.

## 5. Conclusions

The results of this study indicate that mistletoe therapy is safe. While expected effects and mild local or temperature related ADRs were common, severe ADRs (which were mostly temperature related) were uncommon, no serious ADRs occurred, and no ADRs were associated with long-term injury or disability. Observed ADRs were mostly dose-dependent and believed to be related to the immune-stimulating, pharmacological activity of mistletoe. Future research, involving larger numbers of patients, might be important for identifying risk factors for ADRs to mistletoe, especially severe ADRs. Furthermore, continuing research is required to draw conclusions on clinical efficacy of mistletoe therapy.

## Figures and Tables

**Table 1:** Dose levels for different mistletoe products.

	Very low	Low	Moderate	High
Abnoba	≤0.02 mg/mL	>0.02–0.2 mg/mL	>0.2–2.0 mg/mL	>2.0 mg/mL
Helixor	<1 mg/mL	1–<10 mg/mL	10–<30 mg/mL	≥30 mg/mL
Iscador	≤0.01 mg/mL	>0.01–<1 mg/mL	1–<10 mg/mL	≥10 mg/mL
Iscucin	Strengths A and B	Strengths C and D	Strengths E and F	Strengths G and H
Isorel	Strength 1	Strengths 6 and 12	Strengths 24 and 32	Strength 60

**Table 2:** Adverse drug reactions recorded in cancer patients treated with mistletoe extracts.

System organ class (SOC)	Preferred term (PT)	Total patients	Total events	Incidence (%)
Gastrointestinal disorders	Diarrhoea	1	2	0.05
	Nausea	2	2	0.10
	Vomiting	2	2	0.10
*Gastrointestinal disorders total *		5	6	0.26
General disorders and administration site conditions	Chills	4	4	0.21
	Fatigue	1	2	0.05
	Injection site erythema (>5 cm)	22	24	1.14
	Injection site swelling (>5 cm)	4	4	0.21
	Injection site urticaria (>5 cm)	2	2	0.10
	Local reaction (>5 cm)	31	39	1.61
	Malaise	1	1	0.05
	Pyrexia	124	171	6.45
*General disorders and administration site conditions total *		157*	244	8.16
Investigations	Blood glucose increased	1	1	0.05
	Blood pressure decreased	1	1	0.05
*Investigations total *		2	2	0.10
Nervous system disorders	Headache	2	2	0.10
*Nervous system disorders total *		2	2	0.10
Skin and subcutaneous tissue disorders	Pruritus generalised	3	3	0.16
	Rash	2	2	0.10
	Urticaria	4	4	0.21
*Skin and subcutaneous tissue disorders total *		7*	9	0.36
Vascular disorders	Syncope	1	1	0.05
*Vascular disorders total *		1	1	0.05

Total		162*	264	8.42

*This value is not equal to the sum of patients listed for each adverse drug reaction since some patients experienced multiple reactions.

**Table 3:** Odds ratios of predictive factors for experiencing at least one expected effect or adverse drug reaction during mistletoe extract therapy.

	Expected effects		Adverse drug reactions	
	OR (95% CI)	*P* value	OR (95% CI)	*P* value
Female	1.61 (1.11, 2.39)	0.015*	0.95 (0.64, 1.45)	0.821
Age (years)	0.97 (0.96, 0.99)	2.28*e* − 05***	0.99 (0.97, 1.00)	0.110
UICC stage				
0	Reference			
I	0.39 (0.16, 0.97)	0.038*	2.10 (0.41, 38.41)	0.479
II	0.39 (0.16, 0.95)	0.033*	2.00 (0.40, 36.45)	0.505
III	0.26 (0.11, 0.65)	0.003**	2.60 (0.52, 47.43)	0.357
IV	0.15 (0.06, 0.39)	6.11*e* − 05***	2.23 (0.44, 40.72)	0.441

OR: odds ratio; CI: confidence interval; UICC: International Union Against Cancer.

Significance levels: *P < 0.05, **P < 0.01, ***P < 0.001.

**Table 4:** Incidences of adverse drug reactions experienced during mistletoe therapy with concurrent conventional therapies.

Conventional therapies	Number of patients			Incidence (%)
	Included in analysis*	Mistletoe and conventional therapy crossover	ADR during therapy crossover	
Chemotherapy	832	415	10	2.41
Targeted therapies	226	120	1	0.83
Hormone therapy	185	113	1	0.88
Bisphosphates	42	20	0	0.00
Radiation therapy	543	207	5	2.42
Surgery	1371	270	3	1.11

*Only patients that had both start and end dates for mistletoe extract therapy and the appropriate conventional therapy were included in each analysis. ADR: adverse drug reaction.
